# Transtheoretical Psychological Therapy – New Perspectives for Clinical Training and Practice

**DOI:** 10.32872/cpe.13891

**Published:** 2024-04-26

**Authors:** Wolfgang Lutz, Winfried Rief

**Affiliations:** 1 Clinical Psychology and Psychotherapy, University of Trier, Trier, Germany; 2 Clinical Psychology and Psychotherapy, Philipps University Marburg, Marburg, Germany; ??? ???

In this special issue of Clinical Psychology in Europe on "Transtheoretical Psychological Therapy – New Perspectives for Clinical Training and Practice", we focus on new evidence-based transtheoretical concepts of clinical training and practice. A transtheoretical perspective offers a new framework for integrating evidence-based psychological treatment techniques that can have theoretical roots in different theories. Thus, it represents an umbrella that encourages the consideration of all research results and evidence-based treatment proposals, and a fruitful stimulation of insights *across* traditional orientations. Transtheoretical psychotherapy aims to use findings from mechanisms, outcome, process, and feedback research as conceptual frameworks for clinical practice and training.

The history of psychotherapy (now often referred to as *psychological therapy* to include the various newer theoretical concepts) is characterized by a growing number of clinical theories, approaches, and methods with their psychopathological concepts, psychological models of change, and, as a result, the growth of a heterogeneous landscape of professional therapy associations. The major schools of therapy that have emerged from this development have contributed to the establishment of the field and advances in patient care around the world. However, a scientifically sound and clearly defined causal network between mechanisms of change and treatment outcomes has been lacking (Lutz et al., 2022). In addition, service systems around the world are very heterogeneous in the definition and delivery of psychological therapy, and not all treatment concepts applied in clinical practice are scientifically evaluated (Rief et al., 2022).

Simultaneously, an emphasis on treatment approaches and therapeutic schools resulted in inflexibilities and conflicts among colleagues. This frequently resulted in a constricted view of scientific and clinical perspectives, impeding the dynamic advancement of the field in terms of psychotherapy research and finding common ground (e.g. Goldfried, 1980; Hofmann et al., 2022; Lutz et al., 2023). Furthermore, while for example in Germany the official state-wide guidelines prohibit combining procedures in outpatient practice, such combinations are at the same time prevalent in outpatient and inpatient settings both nationally and internationally (Twomey et al., 2023).

In this special issue, transtheoretical concepts and practical implications that go beyond the boundaries of traditional psychotherapeutic procedures and aim for a broader, scientifically based understanding of psychotherapy are discussed. Transtheoretical is understood here as a broader concept, as originally introduced by Prochaska and Di Clemente (1982).

This includes an orientation towards evidence-based treatment procedures and strategies as well as multimethod and multidimensional diagnostic concepts and data-informed decision tools, which can form the basis for transtheoretical and evidence-based clinical training and practice in the future. From our point of view, it is now time to develop broader transtheoretical and scientifically supported concepts for clinical training and practice of psychological therapy, and this special issue wants to contribute to this process.

The papers cover a wide range of issues in the current debate on transtheoretical and integrative concepts as well as specific interventions, clinical methods, or strategies. Several papers present important new developments in transtheoretical metamodels and provide new research- and process-based conceptual frameworks, such as Babl et al. (2024); Boswell et al. (2024); Caspar and Berger (2024); Flückiger et al. (2024); Hofmann and Hayes (2024); Lutz et al. (2024); McNally (2024); Rafaeli and Rafaeli (2024); Rief et al. (2024) and Taubner and Sharp (2024). In addition, several papers discuss transtheoretical clinical case conceptualization and interventions, competencies, and core processes, such as case conceptualization (Gilboa-Schechtman, 2024), homework (Ryum & Kazantzis, 2024), deliberate practice, marker response sequences, and responsiveness (Babl et al., 2024; Boswell et al., 2024), mentalization (Taubner & Sharp, 2024), alliance ruptures (Zilcha-Mano & Muran, 2024), needs and internal self-states (Rafaeli & Rafaeli, 2024), strength (Flückiger et al., 2024), outcome monitoring (Lutz et al., 2024), central processes (Hofmann & Hayes, 2024); disorder-specific knowledge and dynamic networks (Hofmann & Hayes, 2024; McNally, 2024; Rief et al., 2024). While the authors were mostly trained in traditional approaches such as psychodynamic therapy or CBT, they share the intention to search for the common ground of psychotherapy, the basic mechanisms of change, and to foster the development of a common language of psychological treatments. Our vision is a vibrant scientific and practical discipline, optimizing the surplus of integration and progress of treatments, and overcoming artificial boundaries.
